# Effects of an Imposed Flow on Chemical Oscillations Generated by Enzymatic Reactions

**DOI:** 10.3389/fchem.2020.00618

**Published:** 2020-07-23

**Authors:** Oleg E. Shklyaev, Victor V. Yashin, Anna C. Balazs

**Affiliations:** Department of Chemical Engineering, University of Pittsburgh, Pittsburgh, PA, United States

**Keywords:** chemical oscillations, enzymatic reactions, fluid flow, surface-bound enzymes, microfluidic channels

## Abstract

Using analytical and computational models, we determine how externally imposed flows affect chemical oscillations that are generated by two enzyme-coated patches within a fluid-filled millimeter sized channel. The fluid flow affects the advective contribution to the flux of chemicals in the channel and, thereby, modifies the chemical reactions. Here, we show that changes in the flow velocity permit control over the chemical oscillations by broadening the range of parameters that give rise to oscillatory behavior, increasing the frequency of oscillations, or suppressing the oscillations all together. Notably, simply accelerating the flow along the channel transforms time-independent distributions of reagents into pronounced chemical oscillations. These findings can facilitate the development of artificial biochemical networks that act as chemical clocks.

## Introduction

Oscillating chemical reactions in living systems are known to regulate circadian rhythms, varieties of metabolic processes, the transcription of DNA and other important biological functions (Novak and Tyson, 2008; Lim et al., 2013). Within the small-scale dimensions of a biological cell, the diffusion of chemicals is sufficient to ensure the homogeneous mixing of the reagents and therefore, the chemical oscillations are solely functions of time (Elowitz and Leibler, 2000; Novak and Tyson, 2008; Lim et al., 2013; Shum et al., 2015). On a larger spatial scale, when the diffusive homogenization cannot be considered instantaneous, the combination of non-linear chemical reactions and diffusive transport gives rise to chemical Turing patterns (Turing, 1952) and traveling chemical waves (Prigogine and Lefever, 1968). The behavior of the spatio-temporal pattern formation can be adequately described by coupled reaction-diffusion equations. The introduction of an externally imposed flow, however, will modify the chemical fluxes produced by the reaction-diffusion processes and hence, will not only alter the dynamics of the system, but could also provide an effective means of regulating the oscillatory behavior within the solution. Here, we probe how an externally imposed flow affects the chemical oscillations due to coupled enzymatic reactions within a fluid-filled, millimeter sized channel and show that characteristic features of the oscillatory behavior are highly sensitive to the velocity of the applied flow fields.

The chemical oscillations in our systems result from interactions between two enzyme-coated patches, which are localized on the bottom wall of a fluidic chamber. These enzymatic reactions involve two steps. The product of the first enzymatic reaction acts as a promoter for the second reaction. On the other hand, the product of the second reaction acts as an inhibitor for the first. These promoting and inhibiting signals enable the system to exhibit both the positive and negative feedback loops that enable the chemical oscillations. The imposed pressure-driven flow will affect the transport of the reactants between the enzyme-coated patches and hence can alter oscillatory behavior produced by the feedback loops. We also anticipate that the overall dynamic behavior and chemical oscillations in this system will depend on the relative positions of the catalyst patches within the channel.

In order to test the above hypotheses, we analyze the properties of two distinct examples. In the first example, the promotor and inhibitor enzymes are placed in a periodically alternating pattern; with this assumption, we can model the system within a single, periodic unit cell. In the second example, the enzymes are localized at two specific points within an infinitely long pipe. To study these cases, we develop a one-dimensional analytic model for the behavior of chemical phenomena within a long and narrow channel. To validate the 1D model, we compare the predictions from this analytic model to computer simulations of chemical oscillations occurring within two-dimensional channels. The results of both modeling approaches reveal that the distance between the catalytic patches dictates the existence of the chemical oscillations. Furthermore, the speed of the imposed fluid flows can promote or suppress the chemical oscillations in the system. In particular, we show that the imposed flow can enlarge the region in phase space where the chemical oscillations are stable and increase the frequency of the oscillations.

## Theoretical Model

**Figure 1:**
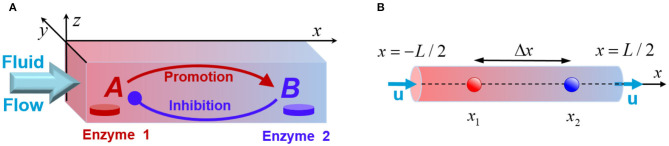
**(A)** Schematic of a fluidic channel that supports chemical oscillations promoted by the enzyme-coated patches, Enzyme 1 and Enzyme 2. The patches initiate transformations of the reactants *A* (red) and *B* (blue), and the fluid flow modifies the chemical kinetics. **(B)** The quasi-1D model of the system that models a channel with the longitudinal size *L* being much greater than the transversal dimensions.

We consider a mixture of chemicals transported along a narrow channel, which has a rectangular cross-section of size *L*_*y*_ × *L*_*z*_, and a long-axis pointing in x-direction, as shown in Figure 1A. The solution contains a number of reactants, but only the two key species, *A* and *B*, are essential for producing chemical oscillations in the system. Specifically, in the presence of a flowing solution that contains the substrate *S*, the immobilized enzymes *E*_1_ and *E*_2_ (see Figure 1A) catalyze the chemical reactions $S\overset{E_{1}}{\to }A$ and $S+A\overset{E_{2}}{\to }B$. In addition to the latter reactions, the species *A* and *B* undergo deactivation over time. We assume that the concentrations of the reactant substrate *S* are constant (Prigogine and Lefever, 1968), and neglect the reverse reactions. Experimentally, this system could be realized in a continuous flow reactor. It is important to note that our theoretical model does not provide an explicit description of all chemical transformations possible in the system. Instead, we design a minimal model that takes into account only the processes that involve the two key reactant species, *A* and *B*.

The chemical transformations of the reagents *A* and *B* can be viewed as a simplified model of the biosynthesis of glutathione that occurs as a two-step process (Jez and Cahoon, 2004; Jez et al., 2004) in all living organisms. During the first step, glutamate-cysteine ligase (GCL) catalyzes production of γ-glutamylcystein from glutamate, cysteine, and ATP. At the second step, glutathione synthetase (GS) catalyzes the formation of glutathione from γ-glutamylcystein, glycine, and ATP. The two-step process (Jez and Cahoon, 2004; Jez et al., 2004) can be expressed as

$$\begin{array}{l} \text{L-glutamate }+\text{ L-cysteine }+\text{ ATP }\overset{\text{GCL}}{\to }\\ \;\gamma \text{-glutamylcysteine + ADP}(R1) \end{array}$$

$$\begin{array}{l} \gamma \text{-glutamylcysteine}\;\text{+}\;\text{glycine}\;\text{+}\;\text{ATP}\;\overset{\text{GS}}{\underset{\;}{\to }}\\ \;\text{glutathione}\;\text{+}\;\text{ADP}\left(R2\right) \end{array}$$

In the living cells, there are mechanisms that maintain concentrations of chemicals within certain range necessary for proper functioning. To mimic the self-regulation in the biological process, we assume that γ-glutamylcystein promotes the production of glutathione, while glutathione inhibits the production of γ-glutamylcystein. Identifying chemicals *A* and *B* with γ-glutamylcystein and glutathione, respectively, and the enzymes GCL and GS with E1 and E2, respectively, we use Michaelis-Menten type reaction rates to realize the proposed regulation mechanism. The substrate for the reaction contains a mixture of all the other components including L-glutamate, L-cystein, glycine, ATP, and ADP; this allows us to represent the reactions (R1) and (R2) as $S\overset{E_{1}}{\to }A$ and $S+A\overset{E_{2}}{\to }B$. Note, that unlike the cell environment where the enzymes GCL and GS are mixed throughout the solution, in our case the enzymes are immobilized at the two surfaces, allowing us to spatially separate the two chemical reactions and, ultimately, generate chemical oscillations.

We note, however, that the proposed reaction scheme is a model that enables us to study the response of chemical oscillations to the advective chemical flux. Because the latter response depends on the relative contribution of the diffusive and advective fluxes, which transport chemicals throughout the solution, the effect should apply to a range of catalytic reactions that promote chemical oscillations by localized catalysts.

The behavior of the system, characterized by the concentrations *C*^*A*^ and *C*^*B*^ of the reagents *A* and *B*, and the fluid velocity **u** = (*u*_*x*_, *u*_*y*_, *u*_*z*_), can be described by the continuity, Navier-Stokes (in the Boussinesq approximation Chandrasekhar, 1961), and reaction and diffusion equations

(1) $$\begin{array}{l} \nabla \cdot \text{u}=0, \end{array}$$

(2) $$\begin{array}{l} \partial_{t}\text{u}+\left(\text{u}\cdot \nabla \right)\text{u}=-\frac{1}{\rho }\nabla p+\nu \nabla^{2}\text{u}, \end{array}$$

(3) $$\begin{array}{l} \partial_{t}C^{j}+\left(\text{u}\cdot \nabla \right)C^{j}+\gamma^{j}C^{j}=D^{j}\nabla^{2}C^{j},\;j=A,B. \end{array}$$

Here and in what follows, ∂_*y*_ is the derivative with respect to a variable *y*, ∇ is the spatial gradient operator, ρ is the density of solution, ν is the kinematic viscosity, γ^*j*^ is the deactivation (decay) rate constant, and *D*^*j*^ is the diffusivity of respective reactants *C*^*j*^, *j* = *A, B*. We assume that the fluid flow with the velocity **u** = (*u*, 0, 0) in the *x*-direction along the channel is generated by the pressure gradient ∇*p* = (*f*, 0, 0) created by an external fluidic pump. For simplicity, we assume that the system is uniform in the *y*-direction and develop a 2D model descibed by *x* and *z* spatial variables.

The chemical reactions, which occur due to the enzyme-coated patches localized on the bottom wall of the channel at *z* = 0 (see Figure 1A), are introduced through the boundary conditions:

(4) $$\begin{array}{l} \;\{z=0,x_{1}-\frac{\delta x}{2}\leq x\leq x_{1}+\frac{\delta x}{2}\}:\\ -D^{A}\partial_{z}C^{A}=k_{1}\sigma_{1}F_{1}\left(C^{B}\right),\;D^{B}\partial_{z}C^{B}=0, \end{array}$$

(5) $$\begin{array}{l} \;\{z=0,x_{2}-\frac{\delta x}{2}\leq x\leq x_{2}+\frac{\delta x}{2}\}:\\ \;-D^{A}\partial_{z}C^{A}=k_{2}\sigma_{2}F_{2}\left(C^{A}\right),D^{B}\partial_{z}C^{B}=k_{2}\sigma_{2}F_{2}\left(C^{A}\right). \end{array}$$

Here, the patch α, where α = 1, 2, is centered at *x*_α_ and coated with the enzyme α, at a surface density of σ_α_. Each patch has length δ*x*. The enzymes are characterized by the reaction rate constants *k*_α_. The functions $F_{1}\left(C^{B}\right)$ and $F_{2}\left(C^{A}\right)$ describe the concentration dependence of the inhibited and promoted reactions, respectively, and are chosen to mimic those for the glutathione biosynthesis pathway (Jez and Cahoon, 2004; Jez et al., 2004):

(6) $$\begin{array}{l} F_{1}\left(C^{B}\right)=\frac{1}{1+{\left(C^{B}/K^{B}\right)}^{n_{1}}},F_{2}\left(C^{A}\right)=\frac{{\left(C^{A}/K^{A}\right)}^{n_{2}}}{1+{\left(C^{A}/K^{A}\right)}^{n_{2}}}, \end{array}$$

where *K*^*B*^ and *K*^*A*^ are the respective inhibition and dissociation constants. As seen from Equations (4) to (6), the rate of production of the chemical *A* decreases with an increase in the concentration *C*^*B*^ (inhibition), whereas an increase in *C*^*A*^ increases the rate of production of *B* until saturation (promotion). Note that the reaction rates in Equations (4)–(6) are taken to be dependent on the cooperativity parameters (Hill coefficients) *n*_α_ > 0, α = 1, 2. Cooperativity of the enzymatic reactions is known to affect the dynamic regimes that could exist in the system (Elowitz and Leibler, 2000; Shum et al., 2015).

Finally, for the solid walls that bound the channel at *z* = 0, and *z* = *H*, we require zero velocity at the walls and zero flux of the reagent concentrations normal to the walls

(7) $$\begin{array}{l} \;\text{u}\left(z=0\right)=\text{u}\left(z=H\right)=0,\\ \partial_{z}C^{j}\left(x,z=0,t\right)=\partial_{z}C^{j}\left(x,z=H,t\right)=0. \end{array}$$

For periodic boundary conditions in the *x*-direction, we set:

(8) $$\begin{array}{l} x:\;\text{u}\left(0\right)=\text{u}\left(L_{x}\right),\;C^{j}\left(0\right)=C^{j}\left(L_{x}\right). \end{array}$$

To simplify the analysis, we reduce the number of model parameters by setting *D*^*A*^ = *D*^*B*^ = *D*, γ^*A*^ = γ^*B*^ = γ, σ_1_ = σ_2_ = σ, and *K*^*A*^ = *K*^*B*^ = *K*. Assuming that our solution is aqueous, we take ν = 10^−6^m^2^s^−1^ and ρ = 10^3^kgm^−3^. We use the glutathione diffusion coefficient (Jin and Chen, 2000) *D* = 0.67 × 10^−9^m^2^s^−1^ to characterize the diffusivity of both reagents *A* and *B*. The deactivation rate γ sets a time and distance (Shklyaev et al., 2020) over which the diffusing reagents turn unto products in the substrate, which we do not model explicitly. To obtain chemical oscillations in a system with a millimeter characteristic length scale, we set γ = 10^−3^s^−1^. The reaction rates of glutamate-cysteine ligase (Jez et al., 2004) (GCL) and glutathione synthetase (Jez and Cahoon, 2004) (GS) were taken as $k_{1}=114\;{\text{s}}^{-1}$, and $k_{2}=3954{\text{s}}^{-1}$, respectively. The inhibition and dissociation constants *K*^*B*^ and *K*^*A*^ both were set to *K* = 3.383 × 10^−2^molm^−3^, which is of the same order of magnitude as the dissociation constants (Jez and Cahoon, 2004) for ATP glycine, and γ-glutamylcystein participating in the reaction Equation (R2). We chose the smallest equal cooperativity parameters *n*_1_ = *n*_2_ = 3 that support the chemical oscillations controlled by the non-linear Hill-type functions presented in Equation (6). Finally, we fix the ratio of the reaction rates *k*_1_σ_1_/*k*_2_σ_2_ = *const* ≈ 0.0288, and use *k*_1_σ_1_ as an independent variable to identify the domain of chemical oscillations and the corresponding values of the enzyme concentrations σ. Note that we obtain enzyme surface densities σ ~10^−7^molm^−2^, which are available through current fabrication techniques.

In what follows, we investigate the behavior of the system controlled by the distance between the enzyme-coated patches Δ*x* = *x*_2_−*x*_1_; reaction rates *k*_1_σ_1_ and *k*_2_σ_2_, which regulate the kinetics of the chemical transformations; and the imposed fluid velocity *u*, which controls the flux of the chemicals $D\partial_{x}C^{j}+uC^{j}$. For this purpose, the system behavior is characterized by the group of parameters (Δ*x, k*_1_σ_1_, *u*).

## Quasi 1D Approximation

When the transversal dimensions *L*_*y*_ and *L*_*z*_ of the channel are much smaller than the characteristic longitudinal scale *L*_*x*_ as schematically shown in Figure 1A, the problem can be reduced to a quasi-one-dimensional system described by a single coordinate *x* (Figure 1B). In this approximation, the externally imposed fluid flow that transports the solution along the channel is characterized by a constant velocity *u*. Appropriate averaging of Equation (3) under the boundary conditions given by Equations (4) and (5) yields the following set of one-dimensional (1D) reaction-diffusion equations:

(9) $$\begin{array}{l} \partial_{t}C^{A}+u\partial_{x}C^{A}+\gamma C^{A}=D\partial_{x}^{2}C^{A}+\frac{k_{1}\sigma_{1}}{H}F_{1}\delta \left(x-x_{1}\right)-\\ \;\frac{k_{2}\sigma_{2}}{H}F_{2}\delta \left(x-x_{2}\right), \end{array}$$

(10) $$\begin{array}{l} \partial_{t}C^{B}+u\partial_{x}C^{B}+\gamma C^{B}=D\partial_{x}^{2}C^{B}+\frac{k_{2}\sigma_{2}}{H}F_{2}\delta \left(x-x_{2}\right). \end{array}$$

The non-linear terms describing the chemical reactions pass from the boundary conditions (Equations 4 and 5), to the right-hand sides of the above 1D equations. We also assume that the spatial extent of the enzyme-coated patches, δ*x*, is much smaller than the length of the channel, *L*_*x*_. Therefore, the location the catalytic patches within the channel and their characteristics are introduced in Equations (8) and (9) by the terms with δ -functions. Finally, the equations are complemented with the periodic boundary conditions

(11) $$\begin{array}{l} x:\;C^{j}\left(0\right)=C^{j}\left(L_{x}\right),\;j=A,B \end{array}$$

For concreteness, we analyze two representative configurations of the channel with specific locations of the enzyme-coated patches. First, we consider an infinite array of alternating enzyme-coated patches distributed equidistantly along an infinite channel. In this case, we solve the problem within a periodic unit cell of length *L*_*x*_ with the neighboring enzyme-coated patches separated by a distance *x*_2_ − *x*_1_ = *L*_*x*_/2 (see Figure 1B). This configuration of the system possesses a symmetry with respect to the velocity reversal from *u* to −*u*. In the second case, we consider only two enzyme-coated patches (1 and 2) separated by a distance *x*_2_ − *x*_1_ and placed within an infinite channel, *L*_*x*_ → ∞. This configuration does not have the degeneracy with respect to the sign change of the fluid velocity. For the both of cases under consideration, we demonstrate that below certain critical values of the reaction rates *k*_1_σ_1_ there exists a time independent solution, whereas the chemical oscillations are possible above the threshold. To find the domain of the oscillatory regime, we solve a relevant stability problem.

## Base State Solution

The equations (Equations 9–11), permit the existence of a time-independent base state, which is governed by the following 1D equations:

(12) $$\begin{array}{l} D\partial_{x}^{2}C_{0}^{A}-u\partial_{x}C_{0}^{A}-\gamma C_{0}^{A}=-\frac{k_{1}\sigma_{1}\delta \left(x-x_{1}\right)}{1+{\left(C_{0}^{B}/K^{B}\right)}^{n_{1}}}+\\ \;\frac{k_{2}\sigma_{2}\delta \left(x-x_{2}\right)}{1+{\left(C_{0}^{A}/K^{A}\right)}^{-n_{2}}}, \end{array}$$

(13) $$\begin{array}{l} D\partial_{x}^{2}C_{0}^{B}-u\partial_{x}C_{0}^{B}-\gamma C_{0}^{B}=-\frac{k_{2}\sigma_{2}\delta \left(x-x_{2}\right)}{1+{\left(C_{0}^{A}/K^{A}\right)}^{-n_{2}}}. \end{array}$$

The solution of Equations (12) and (13) could be presented in a compact form in terms of the Green's function *G*(*x, x*_0_) as

(14) $$\begin{array}{l} C_{0}^{A}\left(x\right)=\frac{k_{1}\sigma_{1}G\left(x,x_{1}\right)}{1+{\left(C_{0}^{B}\left(x_{1}\right)/K^{B}\right)}^{n_{1}}}-\frac{k_{2}\sigma_{2}G\left(x,x_{2}\right)}{1+{\left(C_{0}^{A}\left(x_{2}\right)/K^{A}\right)}^{-n_{2}}}, \end{array}$$

(15) $$\begin{array}{l} C_{0}^{B}\left(x\right)=\frac{k_{2}\sigma_{2}G\left(x,x_{2}\right)}{1+{\left(C_{0}^{A}\left(x_{2}\right)/K^{A}\right)}^{-n_{2}}}. \end{array}$$

The Green's function *G*(*x, x*_0_) is given by the equation

$$\begin{array}{l} G\left(x,x_{0}\right)={\text{e}}^{V\xi_{0}\left(x-x_{0}\right)}\{\begin{array}{c} c_{1}{\text{e}}^{\xi \left(x-x_{0}\right)}+c_{2}{\text{e}}^{-\xi \left(x-x_{0}\right)},\;x<x_{0},\\ c_{3}{\text{e}}^{\xi \left(x-x_{0}\right)}+c_{4}{\text{e}}^{-\xi \left(x-x_{0}\right)},\;x_{0}\leq x \end{array} \end{array}$$

where $c_{1}={\left(2D\xi \left(1-{\text{e}}^{-\left(\xi +V\xi_{0}\right)L_{x}}\right)\right)}^{-1}$, $c_{2}={\left(2D\xi \left({\text{e}}^{-\left(\xi -V\xi_{0}\right)L_{x}}-1\right)\right)}^{-1}$, $c_{3}={\left(2D\xi \left({\text{e}}^{\left(\xi +V\xi_{0}\right)L_{x}}-1\right)\right)}^{-1}$, and $c_{4}={\left(2D\xi \left(1-{\text{e}}^{-\left(\xi -V\xi_{0}\right)L_{x}}\right)\right)}^{-1}$ with *V* = *u*/(2*Dξ*_0_), $\xi_{0}={\left(\gamma /D\right)}^{1/2}$, and $\xi =\xi_{0}{\left(V^{2}+1\right)}^{1/2}$.

**Figure 2:**
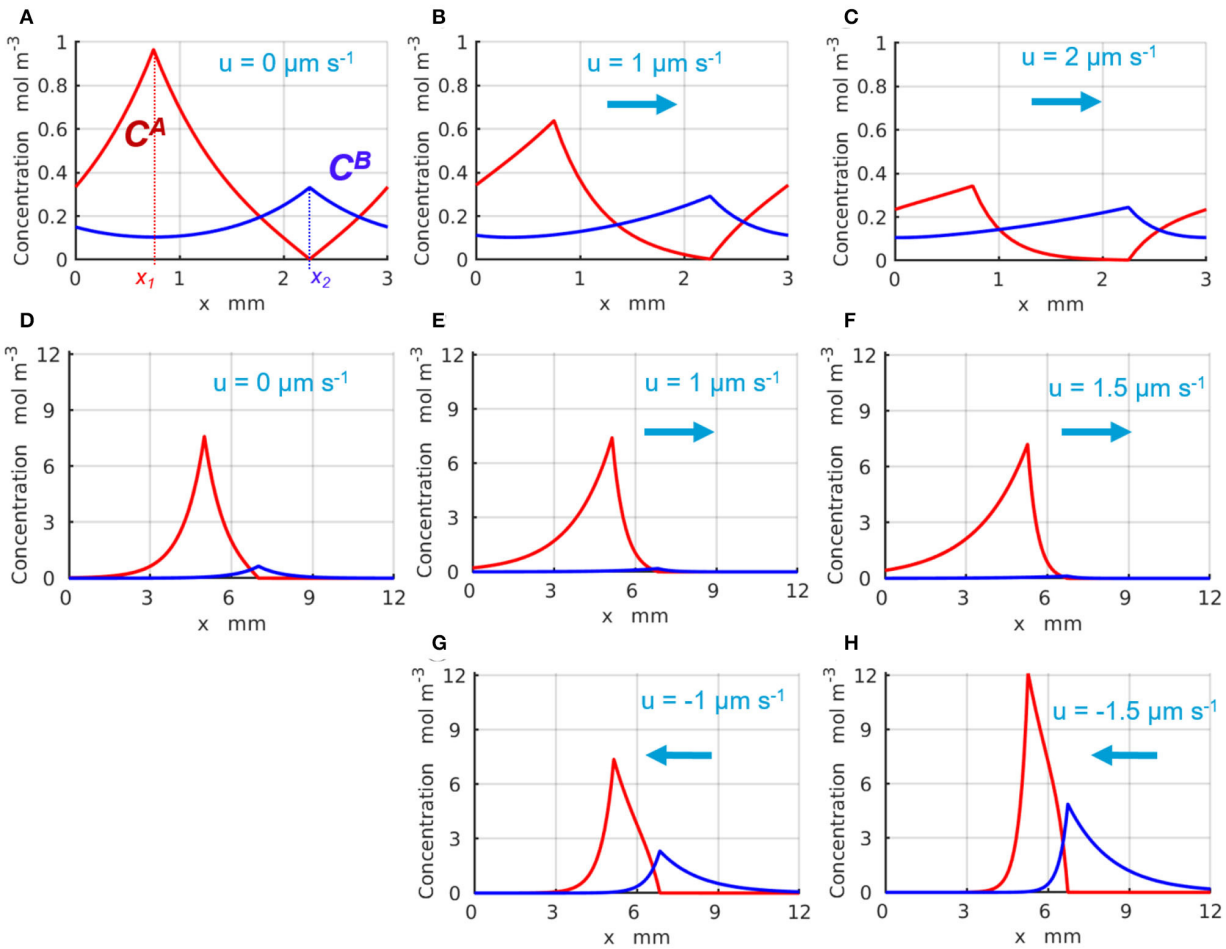
Distribution of the base state concentrations $C_{0}^{A}\left(x\right)$ (red) and $C_{0}^{B}\left(x\right)$ (blue) along the quasi-1D channel. The peaks in $C_{0}^{A}\left(x\right)$ and $C_{0}^{B}\left(x\right)$ occur at the respective locations of the enzymes 1 and 2. For a periodic system **(A–C)** of length *L* = 3mm and the inter-patch distance Δ*x* = 1.5 mm, the concentration profiles are plotted at the fluid velocities *u*= **(A)** 0, **(B)** 1, and **(C)** 2μm·s^−1^. For an infinite system (*L*_*x*_ → ∞), the profiles are shown at **(D)** *u* = 0 μm·s^−1^ and Δ*x* = 2 mm; **(E)** *u* = 1 μm·s^−1^ and Δ*x* = 1.766 mm; **(F)** *u* = 1.5 μm·s^−1^ and Δ*x* = 1.528 mm; **(G)** *u* = −1 μm·s^−1^ and Δ*x* = 1.766 mm; and **(H)** *u* = −1.5 μm·s^−1^ and Δ*x* = 1.528 mm.

The representative time-independent base-state concentration profiles $C_{0}^{A}\left(x\right)$ and $C_{0}^{B}\left(x\right)$ of the reactants *A* and *B* are shown in Figure 2 with the red and blue lines, respectively. The production of the reactants *A* and *B* appears as spikes in the profiles of $C_{0}^{A}$ and $C_{0}^{B}$ around *x*_1_ and *x*_2_, respectively, where the enzyme-coated patches are located. Figures 2A–C illustrate the changes in the chemical concentration profiles in the periodic system with *L*_*x*_ = 3 mm caused by the fluid velocity that increases from *u* = 0 (Figure 2A), to *u* = 1 (Figure 2B), and reaches *u* = 2μms^−1^ (Figure 2C). Figures 2D–H demonstrate the changes in the chemical concentrations that occur in the infinite system, *L*_*x*_ → ∞, as the fluid velocity either increases in the positive direction (of the *x*-axis) from *u* = 0 (Figure 2D), to *u* = 1 (Figure 2E), and to *u* = 1.5μms^−1^ (Figure 2F), or increases in the negative direction to *u* = −1 (Figure 2G) and then to *u* = −1.5μms^−1^ (Figure 2H). The positive fluid velocities (Figures 2E,F) are seen to suppress the spike in the concentration $C_{0}^{B}$ at *x*_2_, whereas the negative velocities (Figures 2G,H) promote the latter. Note that in the infinite system, the concentrations $C_{0}^{A}\left(x\right)$ and $C_{0}^{B}\left(x\right)$ exponentially decay to zero away from the corresponding enzyme-coated patches located at *x*_1_ and *x*_2_ (see Figures 2D–H).

## The Linear Stability Problem

We study the stability of the base state (Equations 14 and 15), by introducing small perturbations *C*^*j*^ = *c*^*j*^(*x*)e^ω*t*^ with a complex growth rate ω = ω_*r*_ + *iω*_*i*_, and linearizing Equations (9) and (10) around the base state. The dynamics of the perturbations is described by the following equations:

(16) $$\begin{array}{l} \partial_{x}^{2}c^{A}-u\partial_{x}c^{A}-\left(\gamma +\omega \right)c^{A}=-k_{1}\sigma_{1}F_{1}^{\prime }c^{B}\delta \left(x-x_{1}\right)+\\ \;k_{2}\sigma_{2}F_{2}^{\prime }c^{A}\delta \left(x-x_{2}\right) \end{array}$$

(17) $$\begin{array}{l} \partial_{x}^{2}c^{B}-u\partial_{x}c^{B}-\left(\gamma +\omega \right)c^{B}=-k_{2}\sigma_{2}F_{2}^{\prime }c^{A}\delta \left(x-x_{2}\right) \end{array}$$

with the periodic boundary conditions $c^{j}\left(0\right)=c^{j}\left(L_{x}\right)$. Here, $i=\sqrt{-1}$ and the primes in ${F_{\alpha }}^{\prime }$, α = 1, 2, denote the derivatives of *F*_α_ with respect to *C*^*j*^. Equations (16) and (17) are solved numerically using the shooting method (Stoer and Burlisch, 1980). The boundary value problem has solutions satisfied by the complex values $\omega \left(\Delta x,L_{x},u,\gamma ,k_{\alpha }\sigma_{\alpha },K^{j}\right)$. The stability curves, (_*k*_1_σ_1_)c_(*u*) vs. Δ*x*, are defined by the condition ω_*r*_ = 0, and separate the domain of the time-independent steady bases states with ω_*r*_ < 0 from the domain of oscillatory regimes, where ω_*r*_ > 0 and ω_*i*_ ≠ 0.

**Figure 3:**
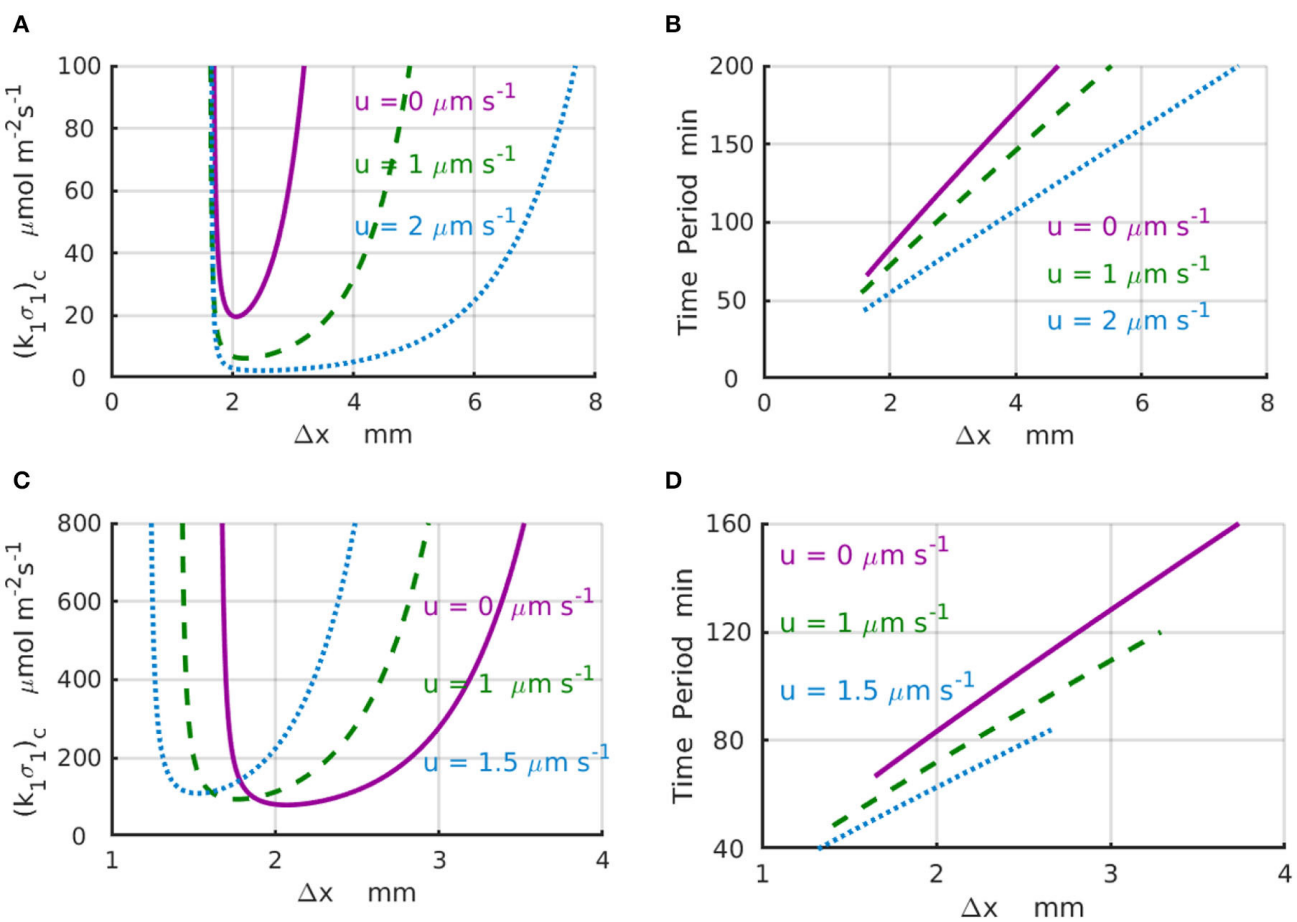
Domain of chemical oscillations for different velocities of the imposed flow. **(A)** Stability curves, (_*k*_1_σ_1_)c_(Δ*x*), for the fluid velocities of *u* = 0 (solid magenta line), 1 (dashed green line), and 2μm·s^−1^ (dotted azure line) for a periodic system with the length of *L* = 4mm. **(B)** Periods of chemical oscillations as a function of the distance Δ*x* between the two enzymes-coated patches obtained along the stability curves in **(A)**. **(C)** Stability curves, (_*k*_1_σ_1_)c_(Δ*x*), for the fluid velocities of *u* = 0 (solid magenta line), 1 (dashed green line), and 1.5μm·s^−1^ (dotted azure line) for an infinite system with *L*_*x*_ → ∞. The respective critical distances between the enzyme-coated patches are Δ*x* = 2, 1.766, and 1.528mm. **(D)** Periods of chemical oscillations as a functions of the distance between the two enzymes obtained along the stability curves in **(C)**.

Results of the linear stability analysis performed for representative values of the imposed fluid velocities are presented in the Figure 3 for a finite system with *L*_*x*_ = 4mm (Figures 3A,B) and an infinite system with *L*_*x*_ → ∞ (Figures 3C,D). In particular, Figure 3A shows the stability curves, (_*k*_1_σ_1_)c_ vs. Δ*x*, calculated for the fluid velocities increasing from *u* = 0 (solid magenta line) to *u* = 1 (dashed green line), and then to *u* = 2μms^−1^ (dotted azure line). The shape of the stability curves demonstrates that the spatial separation Δ*x* = *x*_2_−*x*_1_ between the enzyme-coated patches is a parameter that controls the existence of the chemical oscillations in the system. The periods of the critical chemical oscillations *T* = 2π/|ω_*i*_| for the same velocities are shown in Figure 3B. To illustrate the effect of the imposed fluid flow, we consider a system with Δ*x* = 2mm. An increase in the fluid velocity from zero (solid magenta line) to *u* = 2μms^−1^ (dotted azure line) results in a 5-fold decrease in the critical reaction rate (_*k*_1_σ_1_)c_ required to start the chemical oscillations in the system with Δ*x* = 2mm (Figure 3A). At the same time, the corresponding period of oscillation decreases from *T*(*u* = 0) ≈ 86 min to *T*(*u* = 2μms^−1^) ≈ 56 min (Figure 3B). Note also that the critical distance between the enzyme-coated patches Δ*x*_c_, at which the chemical oscillations first appear at the lowest value of (_*k*_1_σ_1_)c_, is not affected much by the velocity variations.

For the infinite system (*L*_*x*_ → ∞), the stability curves (_*k*_1_σ_1_)c_(Δ*x*) and corresponding plots of the period of the chemical oscillations are shown in Figures 3C,D for the fluid velocities increasing from *u* = 0 (solid magenta line) to *u* = 1 (dashed green line), and then to *u* = 1.5μm s^−1^ (dotted azure line). For a fixed distance between the enzyme-coated patches, Δ*x* = 2mm, the increase of the fluid velocity from *u* = 0 to 1.5μm s^−1^ requires more than a 2-fold increase in the reaction rate in order to surpass the critical value (_*k*_1_σ_1_)c_ needed to excite the chemical oscillations. In contrast with the case of finite system, an increase in the velocity for the infinite system leads to a slight decrease in the critical distance between the enzyme-coated patches from Δ*x*_c_ = 2mm at *u* = 0 to Δ*x*_c_ = 1.77mm at *u* = 1μm s^−1^, and then to Δ*x*_c_ = 1.56mm at *u* = 1.5μm s^−1^. Therefore, in an infinite system, larger reaction rates are required to start the chemical oscillations in the presence of the flow. Simultaneously, the corresponding periods of the oscillation decrease substantially as shown in Figure 3D. In particular, when the fluid velocity increases from *u* = 0 to *u* = 1.5μm s^−1^, the period of oscillation decreases more than twice, namely, from *T* ≈ 86 min at Δ*x*_c_ = 2mm (solid magenta line) to *T* ≈ 40 min at Δ*x*_c_ = 1.56mm (dotted azure line).

**Figure 4:**
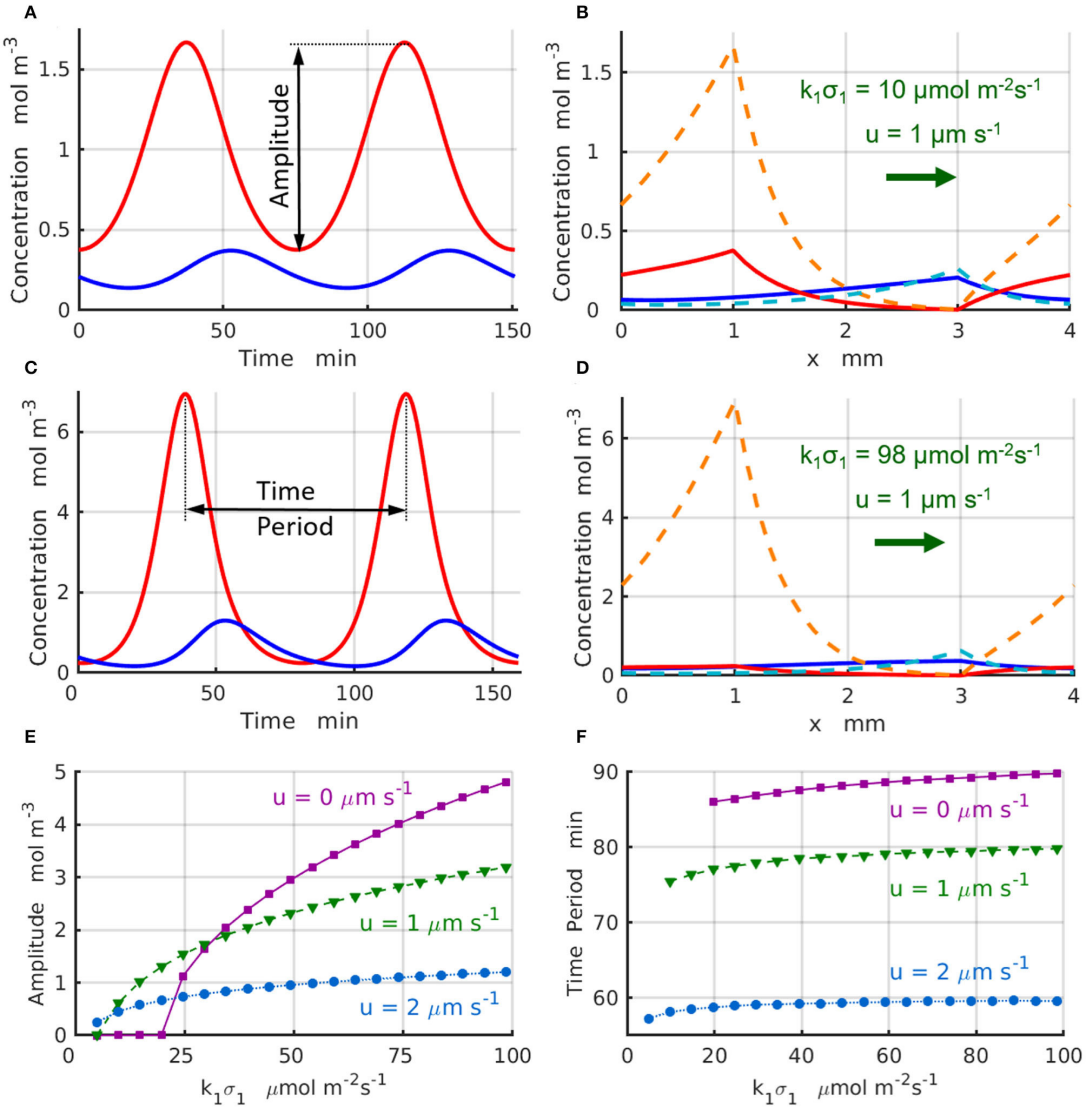
Chemical oscillations in a periodic system with *L*_*x*_ = 4mm and Δ*x* = 2 mm at the supercritical reaction rates, $\mu_{1}^{\;}>\mu_{\text{c}}$. The concentrations $C_{\;}^{A}\left(x_{1},t\right)$ (red) and $C_{\;}^{B}\left(x_{2},t\right)$ (blue) as functions of time at *u* = 1μm·s^−1^, *k*_1_σ_1_= **(A)** 10 and **(C)** 98 μmol·m^−2^s^−1^. Maximal (dashed lines) and minimal (solid lines) values of the concentrations within one period of the oscillation at *u* = 1μm·s^−1^, *k*_1_σ_1_= **(B)** 10 and **(D)** 98μmol·m^−2^s^−1^. **(E)** Amplitude and **(F)** period of the chemical oscillations as functions of the reaction rate *k*_1_σ_1_ for the inter-patch distance Δ*x* = 2 mm at *u*= 0 (solid magenta lines and squares), 1 (dashed green lines and triangles), and 2μm·s^−1^ (dotted azure lines and circles).

The stability analysis reveals that for a fixed reaction rate *k*_1_σ_1_, the chemical instability can occur only within a limited range of distances between the enzyme-coated patches Δ*x*_min_ < Δ*x* < Δ*x*_max_. When *k*_1_σ_1_ < (_*k*_1_σ_1_)c_, the linear stability analysis indicates that the system is in a stable steady state with a time-independent distribution of the concentration profiles $C_{0}^{j}\left(x\right)$ along the channel (Figure 2). At the supercritical reaction rate *k*_1_σ_1_ > (_*k*_1_σ_1_)c_ (Figure 4), the linear stability analysis predicts an instability, at which the concentrations of chemicals *A* and *B*, *C*^*j*^(*x, t*), *j* = *A, B*, exhibit temporal oscillations with a frequency |ω_*i*_|.

The calculations also reveal that depending on the design of the system, the imposed fluid flows can substantially reduce the amount of the enzyme [determined by the critical reaction rate (_*k*_1_σ_1_)c_] required to enable the chemical oscillations in the channel. As well, the flows along the channel can substantially increase the frequencies |ω_*i*_| = 2π/*T* of the chemical oscillation. Moreover, there are conditions, such as at the point Δ*x* = 1.5 mm and $k_{1}\sigma_{1}=200\mu \text{mol}\;{\text{m}}^{-2}{\text{s}}^{-1}$ shown in Figure 3C, when the time-independent chemical distributions at zero flow velocity could be turned into the chemical oscillations by simply accelerating the flow to a velocity *u* = 2μm s^−1^.

The characteristic values of the physical parameters within the instability regions (see Figures 3A,C), where the chemical oscillations exist, determine the relevant time scales Δ*x*/*u*, Δ*x*^2^/*D*, and Δ*xC*_0_/*k*_1_σ_1_ characterizing the rates of advective and diffusive transport, and the reaction rate, respectively. Ratios between these time scales indicate the relative importance of the different mechanisms contributing to the dynamics of the chemical oscillations. For example, the Peclet number, $\text{Pe}=\frac{u\Delta x}{D}$, is defined as the ratio of the diffusive to advective time scales. For a characteristic length scale of Δ*x* = 2 mm, reagent diffusivity of *D* ~ 10^−9^m^2^s^-1^ and fluid velocity of *u*~ 1μms^−1^, the resulting value of Pe~ 2 indicates that the diffusive and advective transport mechanisms are of comparable importance in the system's behavior. On the other hand, the comparison of the stability curves shown in Figures 3A,C for velocities *u* = 0, 1, and 2 μms^−1^, with the corresponding values Pe = 0, 2, and 4, imply that the imposed fluid flow affects chemical oscillations (i.e., noticeably reduces the reaction rate and time period) when the Peclet number is comparable to one.

The relevant diffusive Damkohler number, ${\text{Da}}_{1}^{\text{d}}=\frac{k_{1}\sigma_{1}\Delta x}{DC_{0}}$, is defined as a ratio of the diffusive to reaction time scale, and can be calculated as (_*k*_1_σ_1_)c_ (from Figure 3) multiplied by the factor $\frac{\Delta x}{DC_{0}}\sim \;2.10^{6}$mol^−1^m^2^s (where the scale $C_{0}\sim \;1\;\text{mol}{\text{m}}^{-\text{3}}$ is suggested by the base state solutions in Figure 2). For the given range, $10<{\left(k_{1}\sigma_{1}\right)}_{\text{c}}<10^{3}$ μmolm^−2^s^−1^, in Figure 3, the diffusive Damkohler number varies between the limits $2\cdot 10<{\text{Da}}_{1}^{\text{d}}<2\cdot 10^{3}$. The similarly defined advective Damkohler number, ${\text{Da}}_{1}^{\text{a}}=\frac{k_{1}\sigma_{1}}{uC_{0}}$, varies in the range $10<{\text{Da}}_{1}^{\text{a}}<10^{3}$. The diffusive and advective Damkohler numbers, which are substantially >1, indicate that chemical reactions occur faster than the diffusive and advective mechanisms can transport reagents along the channel between the enzyme-coated patches. This transport-limited scenario for the chemical oscillations provides conditions where the advective flux can significantly amplify the diffusive transport.

## 1D Regimes With Supercritical Reaction Rates

To investigate the system beyond the stability boundaries, we numerically solve Equations (9) and (10) in a 1D cell −*L*_*x*_/2 ≤ *x* ≤ *L*_*x*_/2, with the periodic boundary conditions (Equation 11). We discretize the spatial domain of length *L*_*x*_ into *N*_*x*_ nodes, each representing a cube with a side equal to the grid spacing of d*x* = 100μm, and apply a second order finite difference scheme to integrate the reaction-diffusion equations. Each reaction source term (∝*F*_α_) was modeled as an element of size d*x*. As initial conditions, we use the uniform spatial distribution of reactants $C_{\;}^{j}\left(x,t=0\right)=r^{j}$, where 0 ≤ *r*^*j*^ ≤ 1 is a random number. To match the situations analyzed within the linear stability theory, we perform computations in the domains with two different lengths. The simulations in the short domain, *L*_*x*_ = 4mm, are designed to match the stability analysis developed for the periodically alternating enzyme-coated patches. In these simulations, the chemical processes within one periodic cell affect through the boundary conditions the dynamics of the reactants in the neighboring cells. The simulations in the long domain of *L*_*x*_ = 50 mm ensure the absence of the chemical interactions between the neighboring cells (because the chemical concentrations decay exponentially with the distance away from the enzyme-coated patches) and, therefore, match the prediction of the stability analysis performed for the case of the infinitely long channel with *L*_*x*_ → ∞.

The chemical oscillations, which occur at the supercritical reaction rates *k*_1_σ_1_>(_*k*_1_σ_1_)c_ in the short domain of *L*_*x*_ = 4 mm, are presented in Figure 4. Figure 4A displays the temporal variations of the concentrations $C_{\;}^{A}\left(x_{1},t\right)$ (red line) and $C_{\;}^{B}\left(x_{2},t\right)$ (blue line) that take place at the locations of the enzyme-coated patches *x*_1_ and *x*_2_ for the control parameters *u* = 1 μs^−1^ and $k_{1}\sigma_{1}=10\;\mu \text{mol}\;{\text{m}}^{-2}{\text{s}}^{-1}$. Figure 4B shows maximal (dashed lines) and minimal (solid lines) values of the concentrations $C_{\;}^{A}\left(x,t\right)$ and $C_{\;}^{B}\left(x,t\right)$ achieved during the period of oscillation. Similarly, Figure 4C shows the temporal variations of the reactant concentrations $C_{\;}^{A}\left(x_{1},t\right)$ (red line) and $C_{\;}^{B}\left(x_{2},t\right)$ (blue line), while Figure 4D shows the maximal (dashed lines) and minimal (solid lines) values of the concentrations $C_{\;}^{A}\left(x,t\right)$ and $C_{\;}^{B}\left(x,t\right)$ calculated at the parameters *u* = 1μm s^−1^ and $k_{1}\sigma_{1}=98\;\mu \text{mol}\;{\text{m}}^{-2}{\text{s}}^{-1}$. Comparison of the oscillation dynamics presented in Figure 4A for $k_{1}\sigma_{1}=10\;\mu \text{mol}\;{\text{m}}^{-2}{\text{s}}^{-1}$ and Figure 4C for $k_{1}\sigma_{1}=98\;\mu \text{mol}\;{\text{m}}^{-2}{\text{s}}^{-1}$ reveals that the chemical oscillations at higher reaction rates deviate from the sinusoidal kinetics observed at sufficiently low reaction rates.

To characterize the supercritical regimes of the chemical oscillations, we define the oscillation amplitude of the reactant *A* as $A^{A}=\underset{0\leq t\leq T}{\max}\left(C^{A}\left(x_{1},t\right)\right)-\underset{0\leq t\leq T}{\min}\left(C^{A}\left(x_{1},t\right)\right)$. The amplitudes as functions of the reaction rate $k_{1}^{\;}\sigma_{1}$ are plotted in Figure 4E for the values of fluid velocities increasing from *u* = 0 (solid magenta line and squares) to *u* = 1 (dashed green line and triangles), and then to *u* = 2μms^−1^ (dotted azure lines and circles). The regimes are supercritical and the amplitudes grow approximately in proportion to the square root of the distance from the bifurcation point, $A^{A}\propto {\left(k_{1}\sigma_{1}-{\left(k_{1}\sigma_{1}\right)}_{\text{c}}\right)}^{1/2}$. As seen in Figure 4E, the amplitude of oscillations decreases with an increase in the velocity of the imposed flow. Finally, Figure 4F shows that the period oscillations, *T*, decreases with an increase in both the reaction rate *k*_1_σ_1_ and the fluid velocity. The simulation results projected onto the onset of chemical oscillations are in a good agreement with the critical reaction rates (_*k*_1_σ_1_)c_ predicted by the stability analysis (Figures 3A,B).

**Figure 5:**
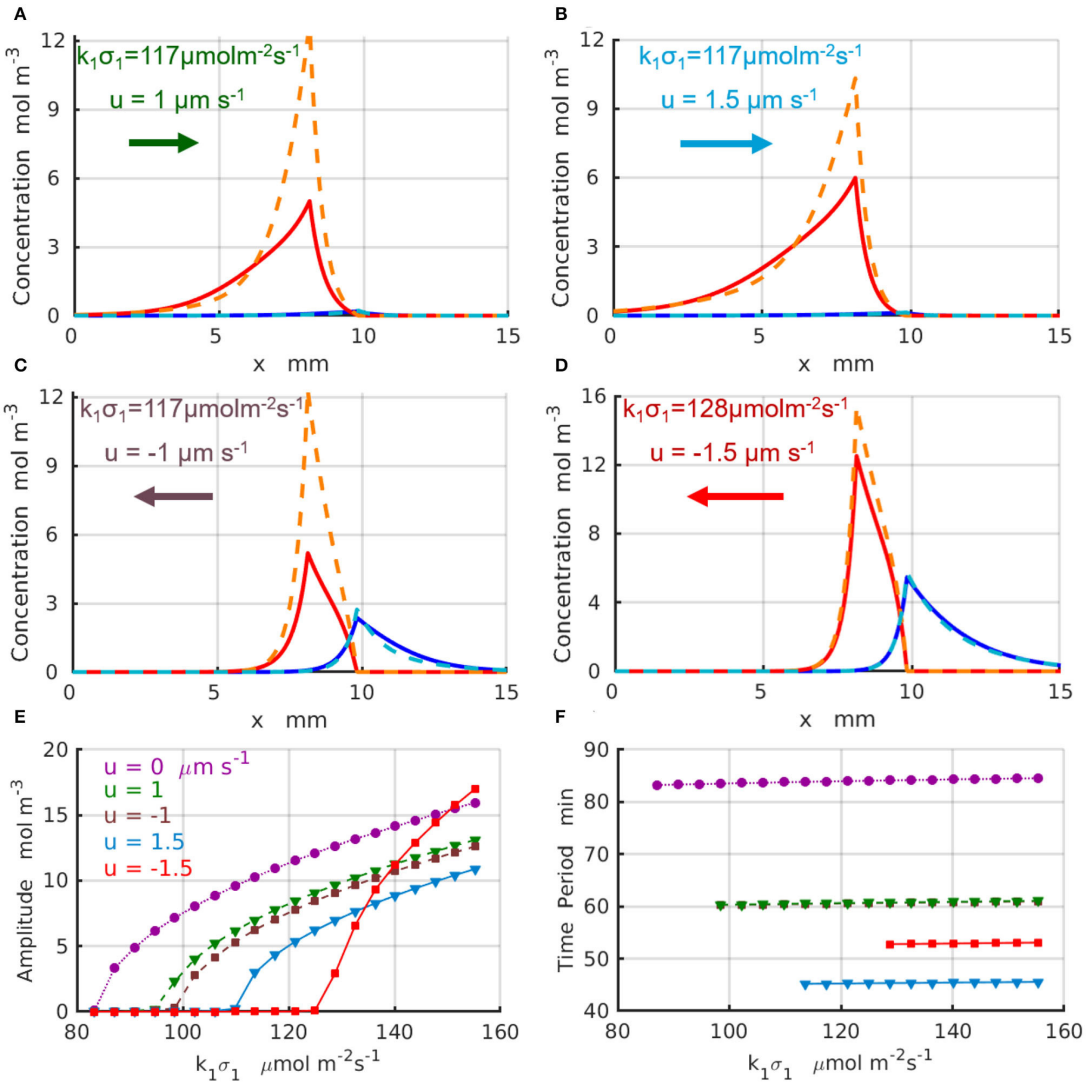
Chemical oscillations in an infinite system with *L*_*x*_ → ∞ at the supercritical reaction rates μ_1_ > μ_c_. Maximal (dashed lines) and minimal (solid lines) concentration profiles *C*^*A*^ (red) and *C*^*B*^ (blue) within one period of oscillations for the set of parameter (Δ*x, k*_1_σ_1_, *u*) **(A)** (1.766mm, 117μmol·m^−2^s^−1^, 1μm·s^−1^); **(B)** (1.528mm, 117μmol·m^−2^s^−1^, 1.5μm·s^−1^); **(C)** (1.766mm, 117μmol·m^−2^s^−1^, −1μm·s^−1^); **(D)** (1.528mm, 128μmol·m^−2^s^−1^, −1.5μm·s^−1^). **(E)** Amplitudes and **(F)** periods of the chemical oscillations as functions of the reaction rate *k*_1_σ_1_ for parameters *u*= 0 and Δ*x* = 2 mm (dotted magenta line and circles), *u* = 1μm·s^−1^ and Δ*x* = 1.766 mm (dashed green line and triangles), *u* = −1μm·s^−1^ and Δ*x* = 1.766 mm (dashed brown line and squares), *u* = 1.5μm·s^−1^ and Δ*x* = 1.528 mm (solid azure line and triangles), and *u* = −1.5μm·s^−1^ and Δ*x* = 1.528 mm (solid red line and squares).

The results for the chemical oscillations catalyzed by two enzyme-coated patches placed in the long simulation domain of *L*_*x*_ = 50 mm are presented in Figure 5. The periodic temporal variations of the concentrations $C_{\;}^{A}\left(x_{1},t\right)$ and $C_{\;}^{B}\left(x_{2},t\right)$ are qualitatively similar to those presented in Figures 4A,C. Figures 5A–D show the maximal (dashed lines) and minimal (solid lines) values of the concentration profiles *C*^*A*^ (red) and *C*^*B*^ (blues) achieved during one period of oscillation; the control parameters are indicated in the figure and specified in the caption. The oscillation amplitudes $A_{\;}^{A}$ as functions of the reaction rate $k_{1}^{\;}\sigma_{1}$ are plotted in Figure 5E for the fluid velocity increasing in the positive direction (of the *x*-axis) from *u* = 0 (dotted magenta line and circles) to *u* = 1μm s^−1^ (dashed green line and triangles), and then to *u* = 1.5μm s^−1^ (solid azure line and triangles). Figure 5E shows the amplitudes for the fluid velocities increasing in the negative direction to *u* = −1μm s^−1^ (dashed brown line and squares) and *u* = −1.5μm s^−1^ (solid red line and squares). At most tested parameter sets, the amplitude of the oscillations decreases with an increase in magnitude of the fluid velocity. In the case of negative velocity of the imposed flow *u* = −1.5μm s^−1^ (solid red line and squares), however, the amplitude of the chemical oscillations increase with an increase in $k_{1}^{\;}\sigma_{1}$ faster than that for the oscillations without fluid flow (dotted magenta line and circles). Finally, Figure 5F shows the period of the oscillations, *T*, which increases with an increase in the reaction rate *k*_1_σ_1_ and decreases with the increasing fluid velocities. In particular, at the fluid flows with velocity *u* = 1.5μm s^−1^ (solid azure line and triangles) the oscillation period, *T* ≈ 46 min, decreases almost twice relative to the case without flow *u* = 0 (dotted magenta line and circles). The simulations projected onto the onset of the chemical oscillations confirm the values of the critical reaction rates (_*k*_1_σ_1_)c_ predicted by the linear stability analysis and presented in Figures 3C,D.

The non-linear 1D simulations reveal that an increase in the frequency of the chemical oscillations under increasing velocities of the imposed flow is in most of the cases accompanied by a reduction of the oscillation amplitude. We found however that there are some parameters and system configurations, for which both the amplitude and frequency of chemical oscillation exhibit a simultaneous increase as indicated by the red lines in Figures 5E,F. Therefore, the design of the system and careful choice of the control parameters, such as the reaction rates and velocity of the imposed flow, are important for tuning the frequency of chemical oscillations to either suppress or amplify the oscillations.

## 2D Chemical Oscillations Under Poiseuille Flow

To test the relevance of the developed 1D model, we compare its predictions with the results of simulations of a more realistic two-dimensional system. We solve Equations (1)–(3) in a periodic 2D unit cell with 0 ≤ *x* ≤ *L*_*x*_, 0 ≤ *z* ≤ *H*. At the solid walls (*z* = 0, *H*) that bound the 2D channel, we require the no-slip conditions for the fluid velocities and zero chemical flux across the parts of the walls free of the enzymes, as described by Equation (7). The periodic boundary conditions in the *x* direction are enforced through the Equation (8). The chemical reactions are catalyzed by the enzymes 1 and 2, which are immobilized at the patches of a finite length δ*x* and are introduced through the boundary conditions given by Equations (4) and (5).

The solution to the Navier-Stokes equation (Equation 2), with an imposed pressure gradient ∇*p* = (*f*, 0, 0) along the channel and the no-slip boundary conditions (Equation 7), on the walls yields the Poiseuille flow, **u** = (*u*_*x*_, 0, 0), with a parabolic velocity profile across the channel, $u_{x}=\frac{f}{2\mu }z\left(H-z\right)$. We use an average across the channel fluid velocity, $u_{\text{a}}=\frac{H^{2}f}{12\mu }$, in order to characterize the effects of the flow on the chemical oscillations, and to compare the obtained results with those of the 1D model controlled by a constant velocity, *u*. For the sake of simplicity, we compare the results obtained for the 1D and 2D models only for the short periodic domain, *L*_*x*_ = 4mm.

In the 2D simulations, the results depend on the length of a patch, δ*x*, in addition to the inter-patch distance Δ*x* and the geometry of the channel described by *L*_*x*_ and *H*. These simulations involve a rectangular domain of size *L*_*x*_ × *H*, which is discretized using a grid 80d*x* × *N*_*z*_d*x* with the grid spacing d*x* = 50μ*m*; the number of nodes in the vertical direction *N*_*z*_ = *H*/dx is defined by *H*. We use the Lattice Boltzmann method to solve the continuity and Navier-Stokes equations (Equations 1 and 2). A second order finite difference scheme is applied to solve the reaction-diffusion equations (Equation 3). Additionally, we use the patches of equal length δ*x* = 0.2mm, and set the distance between them to Δ*x* = 2 mm. The reaction rates are assigned the values $k_{1}\sigma_{1}=98\mu \text{mol}\;{\text{m}}^{\text{-2}}{\text{s}}^{\text{-1}}$ and $k_{2}\sigma_{2}=3403\mu \text{mol}\;{\text{m}}^{\text{-2}}{\text{s}}^{\text{-1}}$.

**Figure 6:**
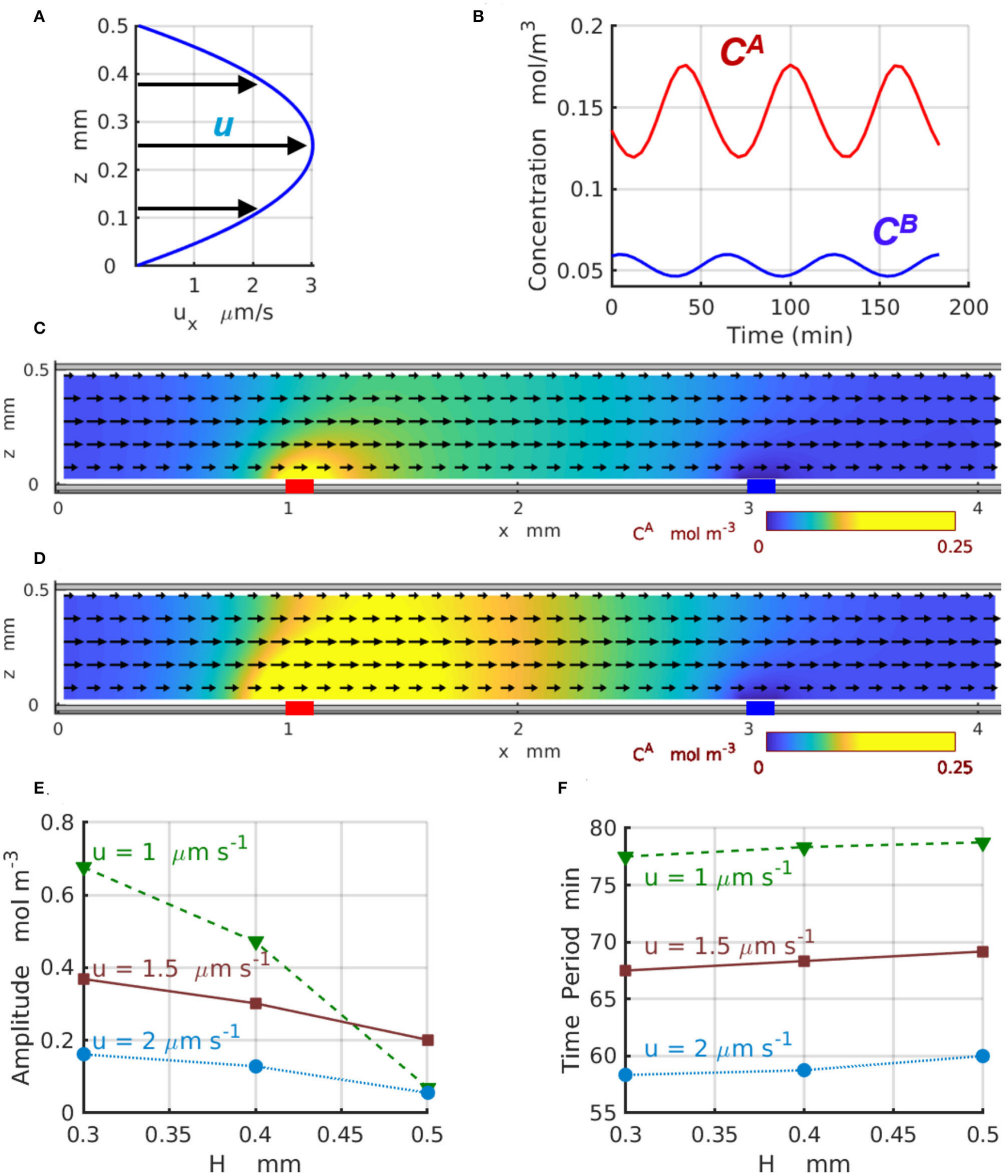
Chemical oscillations in a 2D periodic system with *L*_*x*_ = 4mm. **(A)** Profile of the imposed flow with the averaged velocity $u_{\text{a}}=2\mu \text{m}\cdot {\text{s}}^{-1}$. **(B)** Concentrations $C_{\;}^{A}\left(x_{1},z,t\right)$ (red) and $C_{\;}^{B}\left(x_{2},z,t\right)$ (blue) as functions of time at *z* = 0.1*H*. The spatial distributions of **(C)** minimal and **(D)** maximal concentration *C*^*A*^(*x, z, t*) within one period of oscillation at $u_{\text{a}}=2\mu \text{m}\cdot {\text{s}}^{-1}$ and $k_{1}\sigma_{1}=98\;\mu \text{mol}\cdot {\text{m}}^{\text{-2}}{\text{s}}^{\text{-1}}$. Yellow color indicates regions with high concentrations of reagent *A*. The black arrows show the direction and relative magnitude of the imposed fluid flow. **(E)** Amplitude and **(F)** period of the chemical oscillations as functions of the channel width *H* at the averaged fluid velocity of *u*_a_ = 1 (dashed green line and triangles), 1.5 (solid brown line and squares), and (dotted azure lines and circles).

Figure 6 demonstrates the effect of the imposed flow on the 2D chemical oscillations for channels of different width *H*. In particular, Figure 6A displays the parabolic profile *u*_*x*_(*z*) of the imposed flow for the channel with *H* = 0.5mm and the average velocity $u_{\text{a}}=2\mu \text{m}\;{\text{s}}^{-1}$. Figure 6B shows the temporal variations in the concentrations $C_{\;}^{A}\left(x_{1},z,t\right)$ (red) and $C_{\;}^{B}\left(x_{2},z,t\right)$ (blue) of the reactants *A* and *B*, respectively, calculated at *z* = 0.1*H* for the velocity $u_{\text{a}}=2\mu \text{m}\;{\text{s}}^{-1}$. Figures 6C,D show the 2D distributions of the reactant $C_{\;}^{A}$ (yellow) along the channel corresponding to the maximal (Figure 6C) and minimal (Figure 6D) values achieved within one period of the oscillation (see Figure 6B). Figures 6E,F present the amplitude $A_{\;}^{A}$ and period of the chemical oscillations *T* as functions of the channel height *H* plotted for the three values of averaged velocity of the imposed flow *u*_a_ = 1, 1.5, and 2μm s^−1^ labeled with green triangles, brown squares, and azure circles, respectively. The amplitudes in Figure 6E are calculated as $A^{A}=\underset{0\leq t\leq T}{\max}\left(C^{A}\left(x_{1},z,t\right)\right)-\underset{0\leq t\leq T}{\min}\left(C^{A}\left(x_{1},z,t\right)\right)$ for *z* = 0.1*H*.

The results presented in Figure 6E indicate that for wider 2D channels, the oscillation amplitudes $A_{\;}^{A}$ progressively decrease toward zero. This happens because the geometry of the 2D channels departs from the one-dimensional limit and the discrepancy between 1D and 2D models increases as the channel thickness *H* increases. Due to the difference in the geometry of the channel and enzyme-coated patches, the amplitudes $A_{\;}^{A}$ of the 2D oscillations $C^{A}\left(x_{1},z,t\right)$ calculated at the location *x* = *x*_1_ and *z* = 0.1*H* (in the 2D-domain) are significantly lower than the amplitudes of the 1D oscillations $C_{\;}^{A}\left(x_{1},t\right)$ calculated (in the 1D-domain) for the same reaction rates and presented in Figure 4E. In the agreement with the predictions of the one-dimensional model, the two-dimensional model also shows a reduction in the oscillation amplitude that occurs as the flow velocity increases. At the same time, the period of the 2D chemical oscillations $C_{\;}^{A}\left(x_{1},z,t\right)$, shown in the Figure 6F for the average velocities *u*_a_ = 1 (green triangles) and 2μm s^−1^ (azure circles), is comparable with the period of the 1D oscillations $C_{\;}^{A}\left(x_{1},t\right)$ presented in Figure 4F for the comparable fluid velocities *u*. The oscillation periods within the two models are slightly different because the distance Δ*x* between the enzyme-coated patches in 1D and 2D models are not the same. The period of the 2D-oscillations *T*, shown in Figure 6F, increases with an increase in the channel width *H*, but decreases with the increasing flow velocities what is consistent with the predictions of the 1D model presented in Figure 4F. The dynamics of the 2D chemical oscillations are also presented in the Supplementary Video 1.

## Conclusions

We developed a model to analyze the chemical oscillations produced by enzyme-coated patches in a long, narrow fluidic channel. In contrast to previous models for non-linear chemical dynamics (Scott, 1994; Epstein and Pojman, 1998), we introduced non-linearity into the system through the boundary conditions on the reaction-diffusion equations. The imposed pressure-driven flow along this fluidic channel affects the transport of reagents throughout the fluid and hence, affects the oscillatory behavior in the system. To analyze the effects of the imposed flow, we first described the behavior of the system through a one-dimensional model. The predictions of the 1D model were compared with the results of simulations for two-dimensional channels with a finite thickness. The agreement between the two approaches validates the applicability of the one-dimensional model in capturing the dynamic behavior within the long, narrow channel.

Through our analytical model and simulations, we found that the distance between the enzyme-coated patches dictates the existence of chemical oscillations within the channel. We also identified parameters that control the amplitude and frequency of the chemical oscillations. In particular, we showed that in millimeter-size channels, imposed flows with velocities on the order of 1μm s^−1^ can substantially increase the frequency of the oscillations and modify the range of parameters for which the oscillations occur.

The imposed pressure-driven flow can also significantly reduce the reaction rates needed to produce chemical oscillations by the enzymatic reactions. The flow alters the chemical flux **j** = *D*∇*C*+ **u***C*, which now includes both diffusive and advective contributions to the chemical transport. Additionally, for a range of parameters considered here, the imposed flow reduces the amplitude of the chemical oscillation. Moreover, sufficiently fast flows cause the reagents in the solution to become well-mixed and thereby suppress the oscillations.

These findings elucidate how an externally applied flow affects the chemical oscillations produced by coupled chemical reactions. These results allow us to establish design rules for regulating the dynamics of coupled reaction-diffusion processes and can facilitate the development of chemical reaction networks that act as chemical clocks. Notably, the period of oscillations in biochemical reaction networks (Novak and Tyson, 2008; Lim et al., 2013) is typically on the order of hours. Significantly shorter periods of chemical oscillations can be obtained by combining the localized enzymatic reactions considered here and imposed fluid flows, thereby providing faster chemical clocks for a range of applications.

Finally, we note that instead of utilizing an externally imposed flow, catalytic reactions that generate density variations as reactants are converted to products in fluid-filled chambers can give rise to solutal buoyancy forces, which propel the motion of the fluid through the chambers. As we showed in recent modeling studies, these inherent, chemically-generated flows are also effective at controlling the chemical oscillations in the system (Shklyaev et al., 2020).

## Data Availability Statement

All datasets presented in this study are included in the article/Supplementary Material.

## Author Contributions

OS performed the stability analysis and simulations. VY developed the quasi-1D approximation and identified parameters crucial for the effect. AB organized the work and analyzed the data. All authors contributed to the article and approved the submitted version.

## Conflict of Interest

The authors declare that the research was conducted in the absence of any commercial or financial relationships that could be construed as a potential conflict of interest.

## Supplementary Material

Supplementary Video 1 Oscillations of the concentration of chemical A. Yellow color indicates regions with high concentrations of reagent A. The black arrows show the direction and relative magnitude of the imposed fluid flow. The averaged velocity of the imposed flow is 2 micrometers per second.
